# Efficient and accurate identification of ear diseases using an ensemble deep learning model

**DOI:** 10.1038/s41598-021-90345-w

**Published:** 2021-05-25

**Authors:** Xinyu Zeng, Zifan Jiang, Wen Luo, Honggui Li, Hongye Li, Guo Li, Jingyong Shi, Kangjie Wu, Tong Liu, Xing Lin, Fusen Wang, Zhenzhang Li

**Affiliations:** 1 Department of Otorhinolaryngology, People’s Hospital of Shenzhen Baoan District, Shenzhen, 518101 China; 2 College of Mathematics and Systems Science, Guangdong Polytechnic Normal University, Guangzhou, 510665 China; 3 School of Computer Science and Software, Hebei University of Technology, Tianjin, 300401 China; 4 Department of Pediatrics, First Affiliated Hospital of Guangzhou University of Chinese Medicine, Guangzhou, 510405 China; 5 Cloud & Gene AI Research Institute, Guangzhou, 510635 China; 6 Zhuhai Vocational School of Polytechnic, Zhuhai, 519000 China

**Keywords:** Classification and taxonomy, Image processing, Machine learning

## Abstract

Early detection and appropriate medical treatment are of great use for ear disease. However, a new diagnostic strategy is necessary for the absence of experts and relatively low diagnostic accuracy, in which deep learning plays an important role. This paper puts forward a mechanic learning model which uses abundant otoscope image data gained in clinical cases to achieve an automatic diagnosis of ear diseases in real time. A total of 20,542 endoscopic images were employed to train nine common deep convolution neural networks. According to the characteristics of the eardrum and external auditory canal, eight kinds of ear diseases were classified, involving the majority of ear diseases, such as normal, Cholestestoma of the middle ear, Chronic suppurative otitis media, External auditory cana bleeding, Impacted cerumen, Otomycosis external, Secretory otitis media, Tympanic membrane calcification. After we evaluate these optimization schemes, two best performance models are selected to combine the ensemble classifiers with real-time automatic classification. Based on accuracy and training time, we choose a transferring learning model based on DensNet-BC169 and DensNet-BC1615, getting a result that each model has obvious improvement by using these two ensemble classifiers, and has an average accuracy of 95.59%. Considering the dependence of classifier performance on data size in transfer learning, we evaluate the high accuracy of the current model that can be attributed to large databases. Current studies are unparalleled regarding disease diversity and diagnostic precision. The real-time classifier trains the data under different acquisition conditions, which is suitable for real cases. According to this study, in the clinical case, the deep learning model is of great use in the early detection and remedy of ear diseases.

## Introduction

As is known to us all, the sense of hearing is considered as one of the most important five senses, since the sense of hearing is human lives mainly rely on^1^. However, as a common disease, if not be received early and treated validly, ear disease may leave some negative effects, for example, hearing impairment. In the estimation of ear diseases in the clinic, conventional otoscopy or otoendoscopy is an important component of physical examination at the first step. However, otoscopy or otoendoscopy used in diagnosis can be easily misdiagnosed for non-otolaryngologists^2^. Research of Pichichero, Poole^3^, for example, found that the average accuracy of otitis media diagnosed by 514 pediatricians was only 50%. Such low diagnostic accuracy hinted that, without the assistance of supplementary resources, testing the diagnosis of ear disease will be difficult, even for experts. Based on such a situation, there is a great need to find a new diagnostic strategy to improve diagnostic accuracy.

In these years, deep learning has acted as a promising method for image recognition or classification, is the foundation of automatic image perceiving, processing and deciding, and has been a heated topic in the area of computer vision for a long time^4,5^. Deep learning has been applied in several medical imaging areas, such as the development and validation of a deep learning algorithm for detection of diabetic retinopathy in retinal fundus photographs^6^, large-scale deep learning for computer-aided detection of mammographic lesions^7^. What’s more, deep learning has been widely applied in ear and hearing disease classification^8–10^. In these deep learning applications, deep convolutional neural networks (CNNs)^4^ is playing a very important role in image recognition or classification. Very little prior professional knowledge is needed to input during the training procedure of deep CNNs models. Emerged as a feature extractor, some pre-trained deep CNNs can rival or excel in the execution of domain-specific, handcrafted features^11–14^. Comparing to conventional spectral classifiers, deep CNNs is more accurate in image recognition problems due to the millions of weights with multiple layers but have a high computational cost when training the model^10,15^.

To the best of our knowledge, the purpose of this research is to set up an automatic discriminating system for ear diseases by a deep learning model. The performance of twelve public models was evaluated by accuracy and training-validation time. Based on the assessment results, two best models among 12 models were chosen to build an ensemble classifier which then can design and accomplish a real-time automatic identification of ear diseases system.

## Materials and methods

### Patient selection and data preprocessing

In this study, our dataset was obtained from 41,056 patients who were diagnosed in the department of otolaryngology in the people's hospital of Shenzhen Baoan District, from July 2016 to August 2019. Usually, patients got their eardrums and external auditory canal (EAC) photos via a conchoscope upon visit. These images were got by using standard endoscopes (Matrix E2, XION GmbH, Berlin, Germany) tethered to Olympus CV-170 digital endovision camera systems (Olympus Corporation, Tokyo, Japan). The resolution rate of these images is 586 × 583 pixels. In order to unify graph data and keep the original shape, we uniformly cropped and scaled these images with a size of 448 × 448 pixels with a ratio of 1:1. We chose 20,542 images, about 53.55% of the total candidate images. Male 11,797. female 8,745. occupied 57.43% and 42.57% of the total number of the selected images, respectively, as shown in Table 1. According to the age, aged (0,10] years have the maximum (22.187%) , aged (30,40] years (21.169%) and (20,30] years (20.861%) in order. Simultaneously, those images were randomly split into three sets, which of 80% for training, 20% for validation, respectively. The training set and validation set have no repetition and are consistent with each model that we trained.

**Table 1:** Sample characteristics.

Age	(0,10]	(10,20]	(20,30]	(30,40]	(40,50]
Male	13.451%	4.323%	11.451%	12.612%	8.308%
Female	8.736%	2.391%	9.410%	8.557%	6.055%

Age	(50,60]	(60,70]	(70,80]	(80,90]	> 90
Male	4.322%	2.124%	0.634%	0.194%	0.009%
Female	4.421%	2.266%	0.583%	0.137%	0.015%
Total	Male: 57.43%; Female: 42.57%				

This study confirmed that all methods were implemented in accordance with the relevant guidelines and regulations of the ethics committee of Shenzhen Baoan District People's hospital. It is confirmed that all the experimental protocols have been approved by the ethics committee of Shenzhen Baoan District People's hospital. The informed consent of all subjects was confirmed, and the informed consent of parents and/or legal guardians was obtained for those under 18 years old.

### Labeling of images

**Figure 1:**
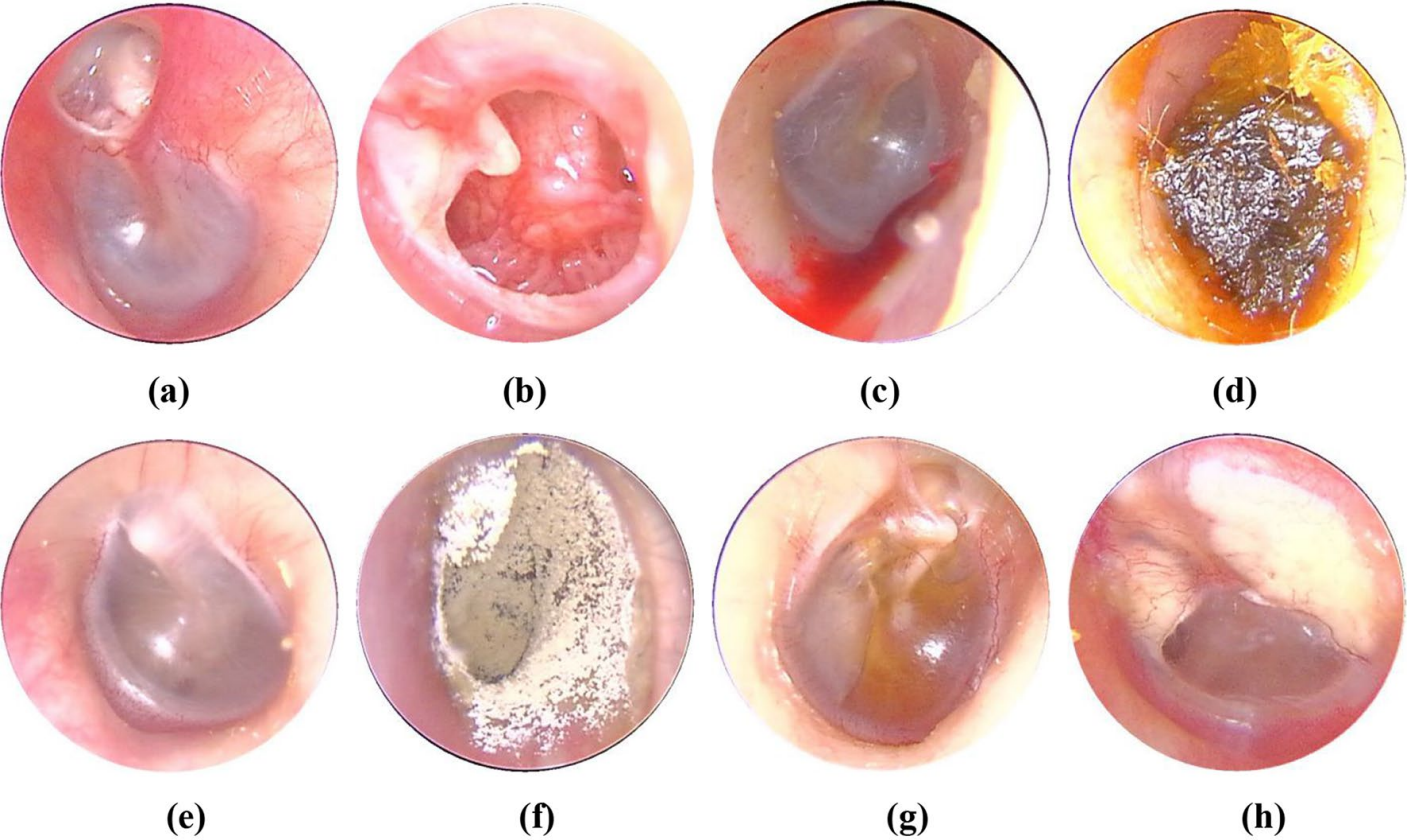
Otendoscopy image and eight diagnostic classes of ear diseases. (**a**) Cholestestoma of middle ear (n = 818). (**b**) Chronic suppurative otitis media (n = 3169). (**c**) External auditory cana bleeding (n = 694). (**d**) Impacted cerumen (n = 5453). (**e**) Normal eardrum (n = 4217). (**f**) Otomycosis external (n = 2256). (**g**) Secretory otitis media (n = 2448). (**h**) Tympanic membrane calcification (n = 1037).

Image samples of eardrums and EAC were divided into eight categories based on the Colour Atlas of Endo-Otoscopy^16^, as shown in Fig. 1. All the image classification was implemented by six ear specialists with more than six years of experience. The number of examples representing each ear disease category is shown in Table 2.

**Table 2:** The number of examples representing each ear disease category.

Category	CME	CSOM	EACB	IC
Number	818	3169	694	5453

Category	NE	OE	SOM	TMC
Number	4217	2256	2448	1037

1. *Normal eardrum and EAC* (included completely normal eardrum, normal with healed perforation or some tympanosclerosis, NE, Fig. 2).

**Figure 2:**
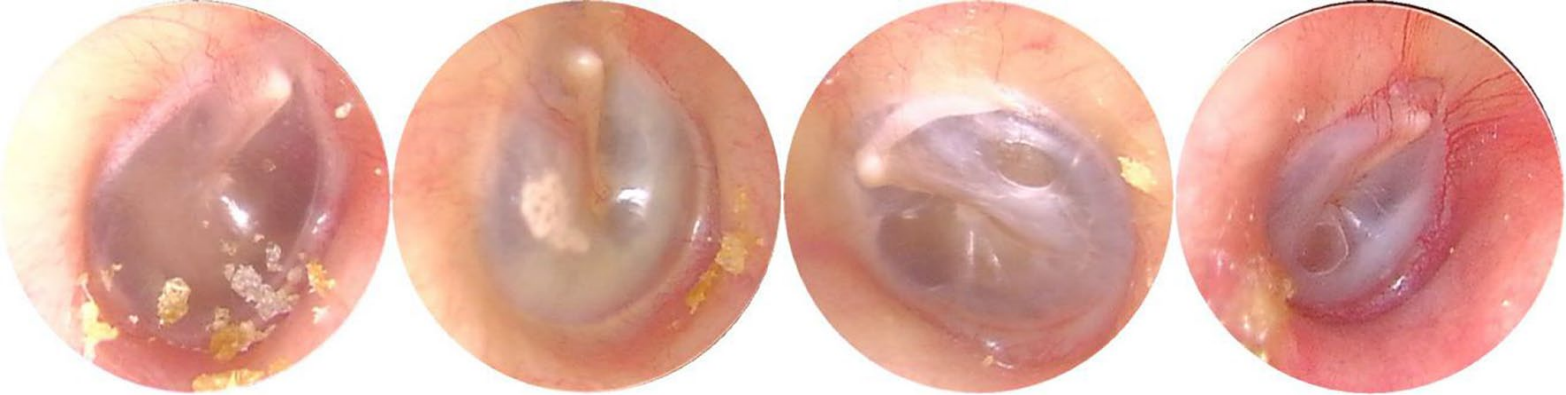
Example of image diversity labelled “Normal”.
2. *Chronic suppurative otitis media (CSOM)* There is perforation of tension of tympanic membrane, and they are not a uniform size. Most of them are single shots. The residual tympanic membrane may have calcification, ulceration and granulation tissue growth around the perforation margin.
3. *Cholestestoma of middle ear (CME)* Loose inner pocket can be seen, and white exfoliated epithelium can be seen inside the pocket.
4. *External auditory cana bleeding (EACB)* Bright red blood can be clearly seen in the external auditory canal.
5. *Impacted cerumen (IC)* The external auditory canal can be blocked by brown-black or yellowish-brown lumps. The cerumen masses have different textures, some are loose like mud; some are hard as stone.
6. *Otomycosis external (OE)* The external auditory canal and tympanic membrane are covered with yellow black or white powdery or villous fungal masses. The short process of the malleus is apparently exoid.
7. *Secretory otitis media (SOM)* The tympanic membrane is invaginated and the handle of the malleus moves backward and upward. When the tympanic cavity has effusion, the tympanic membrane loses its normal luster, showing light yellow, orange oil or amber color, but If the liquid does not fill in the tympanic cavity, the liquid level can be seen through the tympanic membrane.
8. *Tympanic membrane calcification (TMC)* The calcification of the tympanic membrane is deposited like white plaque, which is located in the fibrous layer of the tympanic membrane, but the reason is unclear. It may be related to chronic inflammation, such as chronic otitis media and so on, which can be found in the entire intact and perforated tympanic membrane.

### Training transfer learning network models

In order to extract features from eardrums and EAC white light images for the automated detection of ear diseases, we used a model method which is typically used to solve image classification in computer vision^17,18^. In many models of deep learning models, ResNet^19^ (ResNet50, ResNet101), DensNet-BC^20,21^ (DensNet-BC121, DensNet-BC161, DensNet-BC169), Inception-V3^22^, V4^23^, Inception-ResNet-V2^23^ and MoblieNet-V2^24^, V3^25^ were implemented and compared performance data from release to release, such as Inputting the image samples, training network, optimizing the network model. Traditionally, the feature maps of the last convolutional layer are vectorized and fed into fully connected layers followed by a softmax logistic regression layer. However, fully connected layers are prone to overfitting. In this process, therefore, we used global average pooling in place of the fully connected layers in each model, generating eight output nodes with a softmax activation function. This have some advantage^26^: (I) enforcing correspondences between feature maps and categories, thus the features can easily be interpreted as categories confidence maps. (II) No parameter to optimize in the global average pooling. (III) Global average pooling sums out the spatial information thus it is more robust to spatial translations of the input. The training makes full use of a stochastic gradient descent method^27^ with a batch size of 100, an epoch of 15, an initial learning rate of 0.01, momentum of 0.9 and weight decay of 10^–4^ to optimize parameters. *L*_2_-regularization, dropout and data augmentation were applied to prevent overfitting. The images were flipped horizontally and vertically and then rotated 90 and 180 degrees. Nerves run in various directions, and a change of the nerve direction will not cause any problems.

This study was performed using the deep learning framework PyTorch^28^ through four graphics processing units (Tesla K80, NVIDIA) in Dell T640 station (inc., USA). For data augment in the process of model training, we performed random X and Y flip horizontal and vertical of input images.

Firstly, we classified the features of image samples of eardrums and EAC from the training sets by feeding to the deep neural network in the frame of PyTorch, and then we observed the performance of the training model on the validation dataset, simultaneously. And when the loss and the accuracy were stable, the training was stopped.

#### Model structure adjustment

**Figure 3:**
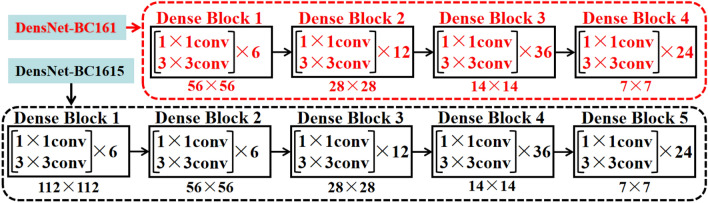
A schematic of model structure adjustment for DensNet-BC1615.

To reduce the size of image features slowly in the convolution operation process of training, we added one dense block ([1 × 1 conv, 3 × 3 conv] × 6) which is the same as the first dense block of DenseNet-BC in the DenseNet-BC^20,21^ framework (the growth rate is *k* = 32) and compression is 0.5). Take DensNetBC161 for example, added one dense block ([1 × 1 conv, 3 × 3 conv] × 6) as the first dense block in DensNet-BC161, and the output size is 112 × 112 (tagged as DensNet-BC1615), as shown in Fig. 3. Others, such as, DensNet-BC121 → DensNet-BC1215, DensNet-BC169 → DensNet-BC1915. Therefore, a total of 12 models were implemented and compared performance data from release to release adding the model described above.

#### Selection of two appropriate models

Through the evaluation of the accuracy and calculation time performance among the 12 models, the appropriate models were selected. 80% and 20% of the images were set up the training set and validation set, respectively, from a total of 20,542 otoendoscopic images. The model optimization step was training-validation which was executed twice with training and validation set respectively.

#### Ensemble classifier

An ensemble classifier was constructed by combining classifiers' outputs of the two appropriate models. Each classifier model assigns the probability of an input image to eight tags (NE, CME, CSOM, EACB, IC, OE, SOM, TMC) and the maximal probability among all tags is considered as a predictable label. The ensemble classifier combines 8-term score vectors from the predicted results of the two models together and the class with a maximal score will be treated as the final forecast image's label.

#### Sensitivity–specificity curve

Sensitivity and specificity are frequent clinimetric parameters that together define the ability of a measure to detect the presence or absence of a specific condition (i.e., likelihood ratio). On the whole testing set, a population-level sensitivity and specificity were calculated based on the following formula.

$$ Sensitivity = \frac{TP}{{TP + FN}}, $$

$$ Specifificity = \frac{TN}{{TN + FP}}, $$

where *FP*, *PN*, *TP* and *PN* represent the numbers of false positives, false negatives true positives and true negatives, respectively. A sensitivity–specificity curve can be created by changing the threshold value *t* (probability *p* ≥ *t*, where *t* is a threshold value).

#### Confusion matrix

We used the confusion matrix to evaluate the quality of the output of the classifier. The values in the diagonal line represent the number of correctly predicted samples, while the values not in the diagonal elements represent the number of misclassified samples. If the diagonal values are very high, it indicates the classifier has a very good performance.

#### Overall accuracy

The overall accuracy is the ratio of the number of correctly categorized images to the total number of testing images, as shown below:

$$ Overall{\kern 1pt} {\kern 1pt} {\kern 1pt} Accuracy{ = }\frac{{N_{{correctly{\kern 1pt} {\kern 1pt} {\kern 1pt} classified{\kern 1pt} {\kern 1pt} {\kern 1pt} images}} }}{{N_{{testing{\kern 1pt} {\kern 1pt} {\kern 1pt} images}} }} $$

## Results

### Model and performance analysis

The number of parameters, training and validation time (Model optimization time) and accuracy of every transferred model were revealed in Table 3. The number of parameters was calculated by user-defined python programs, as shown below.

**Table 3:** Performance table of training models.

Transferred models	Accuracy	GPU time (s)	Parameters	Processing time (s)
MoblieNet-V2	93.455	27,240	2,235,200	0.0374
MoblieNet-V3	93.884	24,758	2,946,622	0.0357
Inception-V4	93.000	98,270	42,681,353	0.1309
ResNet50	93.581	51,098	25,557,032	0.0668
ResNet101	93.632	78,844	42,516,552	0.1099
Inception-ResNet-V2	94.617	111,849	54,318,760	0.1604
DensNet-BC121	94.188	54,192	6,962,056	0.0859
DensNet-BC161	94.564	78,453	26,489,672	0.1707
DensNet-BC169	94.541	56,477	12,497,800	0.1090
DensenetBC1215	94.364	56,079	7,548,920	0.0809
DensenetBC1615	95.099	80,895	27,893,456	0.4512
DensenetBC1695	94.339	58,209	13,122,040	0.1318
Ensemble	–	–	–	0.5708

GPU time is the processing power needed for training and validation the model. Processing time means the time of each model to identify the same input image.

*def get_model_parameter_num(net):*

*total_num = sum(p.numel() for p in net.parameters())*

*trainable_num = sum(p.numel() for p in net.parameters() if p.requires_grad)*

*return {'Total Time': total_num, 'Train Time': trainable_num}*

**Figure 4:**
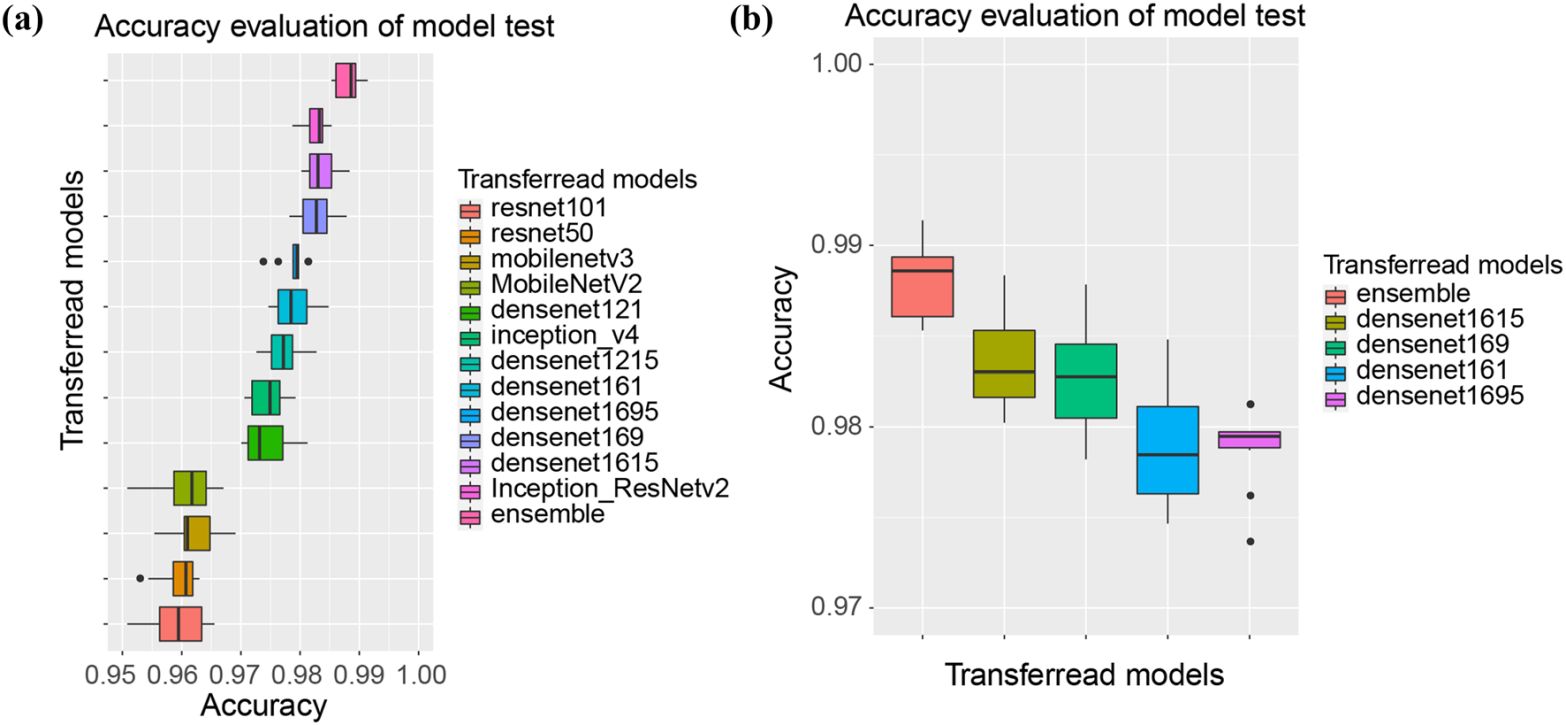
The accuracy comparison between different models on 10 sampling datasets.

The hidden layer of the models had no significant improvement. There is not a hidden layer in the fully connected layer of the ten models. Calculating results show that the best accuracy was the DenseNet-BC1615 (95.099%), followed by Inception-ResNet-V2 (94.617%), DenseNet-BC161(94.564%) and DenseNet-BC169 (94.541%), as shown in Table 3. For model optimization time, DenseNet-BC1615, Inception-ResNet-V2, DenseNet-BC161 and DenseNet-BC169 were 80,895, 111,849 and 78,453 and 56,477 s, respectively. In order to test the stability of the model, the 12 models which we have been trained were evaluated by 10 percent of the sample which collected randomly from the training set. Repeated the previous step ten times and recorded its accuracy, and then and then performed repeated measures one-way ANOVA, as shown in Fig. 4a. One-way ANOVA indicated that the DensNet-BC1615 and DensNet-BC169 increased more significantly than other models except for the Inception-ResNet-V2 (the reason it was not chosen was that it took too long to train). Then we analyzed the significance of the two models we selected and the best two of the remaining models. It further showed that the two models increased diagnostic accuracy significantly (p-value = 2.74e−09), as shown in Fig. 4b.

**Figure 5:**
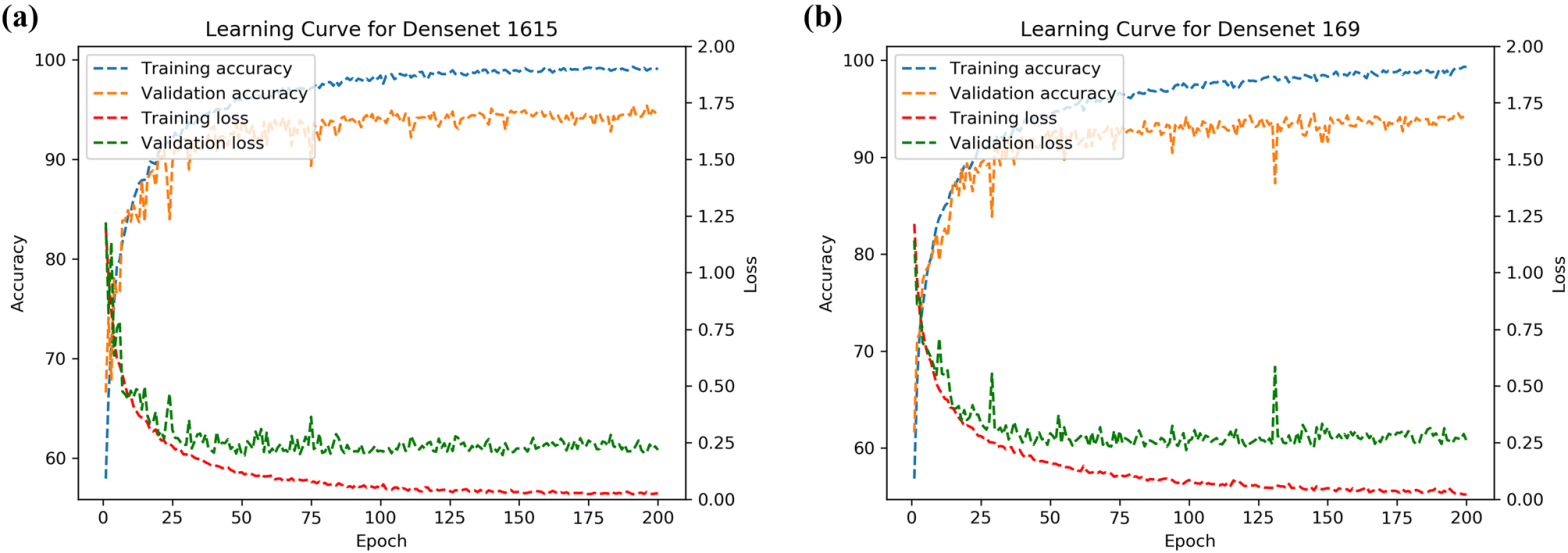
The learning curves of 2 selected models, DenseNet 1615 (**a**) and DenseNet 169 (**b**).

Together with the accuracy and the model optimization time, DenseNet-BC1615 and DenseNet-BC169 were selected to be the best-transferred network models to form an ensemble classifier. To better display the training process, we add learning curves to visualize the training performance of these 2 selected deep learning models (Fig. 5).

**Figure 6:**
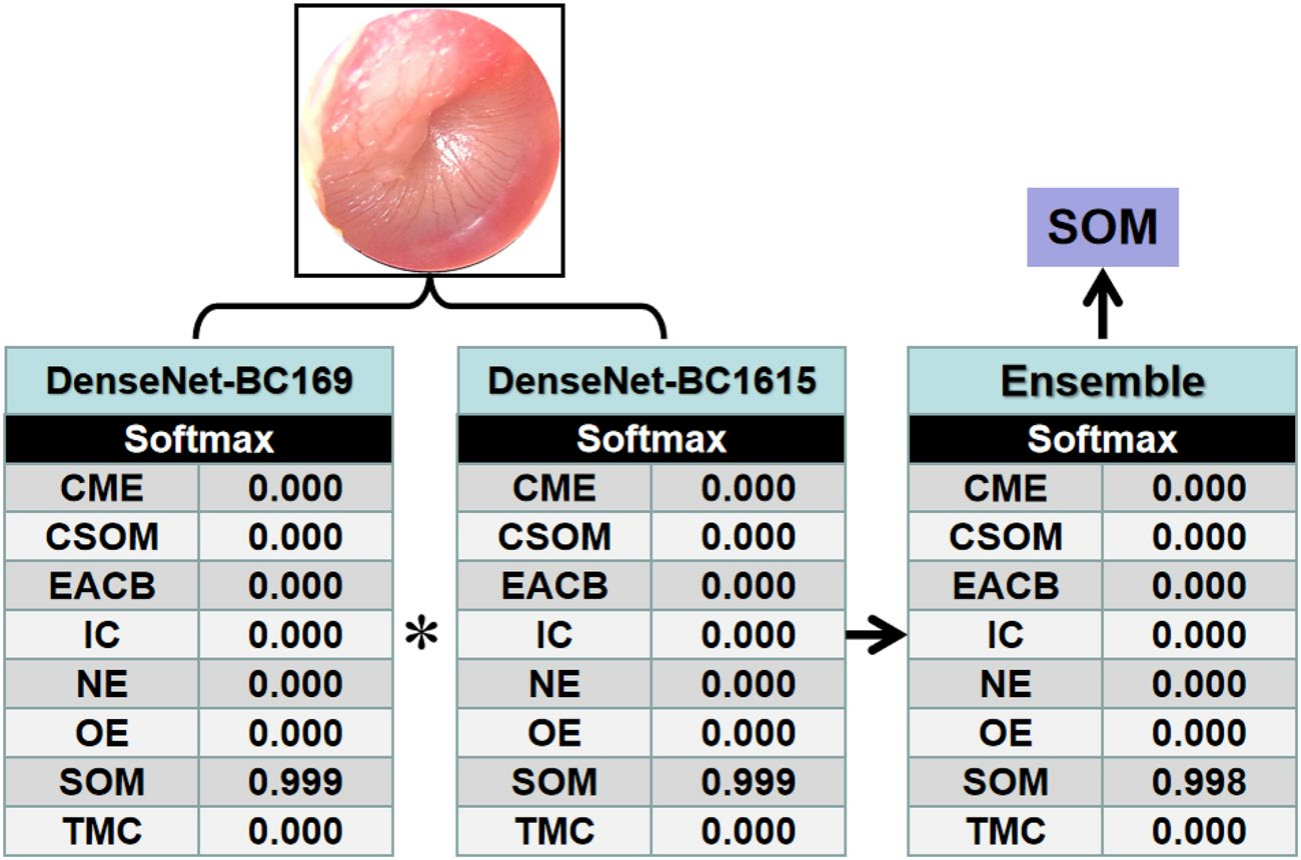
A sketch of structure and an operating diagram for the ensemble method classifies given otoendoscopic image.

From the two models, the classification mechanism of the ensemble classifier is estimated according to the following formula.

1 $$ S = {\text{ max}}\{ x|x \in S_{{1}} *S_{{2}} \} $$

where *S*_1_ and *S*_2_ are the lexicographic vector of an input image predictor score of the two models, respectively. *S*_1_*S_2_ represents the dot product of the *S*_1_ and *S*_2_ vectors. *S* is a predicted label score of the ensemble classifier. For this design, we also give an example of the ensemble classifier running results which were shown in Fig. 6.

**Figure 7:**
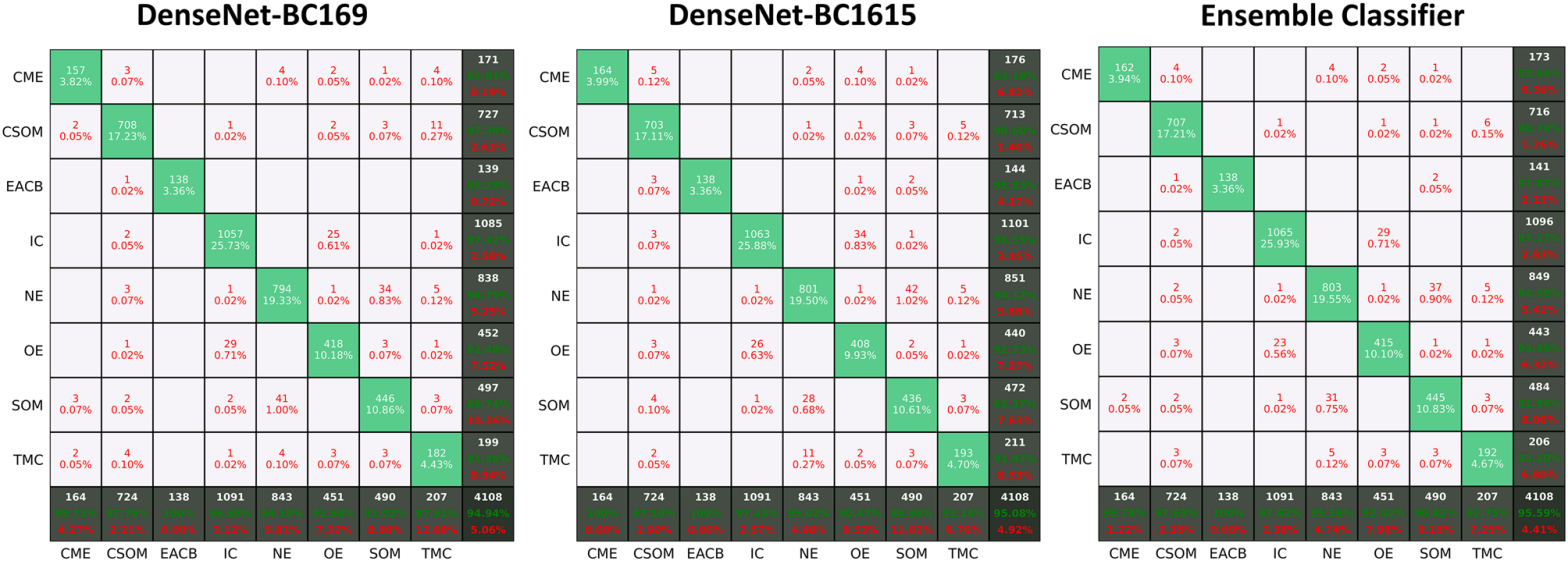
Confusion matrices for DensNet-BC169, DensNet-BC1615, and ensemble classifier at the test sample sets having a maximal accuracy.

In this research, we calculated the confusion matrices for DensNet-BC1615, DensNet-BC169 and the ensemble classifier that were shown in Fig. 7. These results are obtained by identifying the testing data with these 3 models. The testing data covers 10% of total images and is not used during the whole training process. The average classification accuracy was obtained on DensNet-BC1615, DensNet-BC169 and the ensemble classifier reached 94.94%, 95.08%, and 95.59%, respectively. The combined forecasting method possessed the two single method's advantages, and the result showed that forecast value was more accurate.

**Figure 8:**
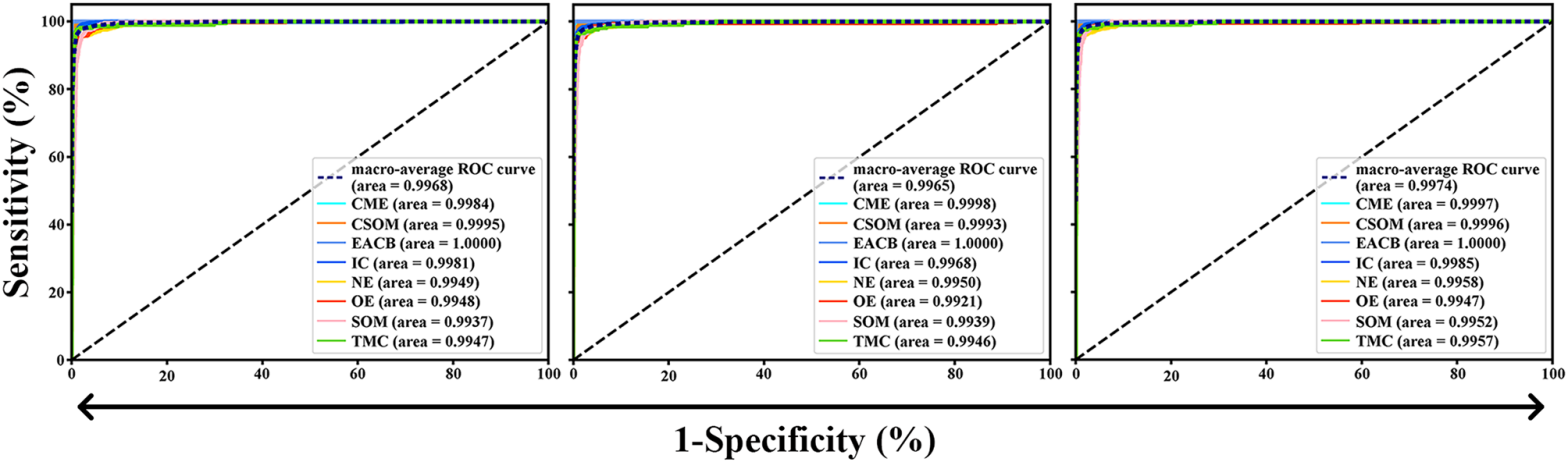
The sensitivity–specificity curve of the ensemble classifier at the test sample sets for Urgent versus Non-urgent binary classification.

We assessed the ensemble classifier according to the performance of generating binary predictions on Urgent versus Non-urgent subjects. Blood transfusion is the means of life-saving treatment of many diseases. Such a binary classification task has been very important for clinical significance since emergency cases should be treated immediately. Any delay, attributed to misclassification, for example, will increase the risk of death. The performance of the Urgent versus Non-urgent based on the sensitivity–specificity curve can be clearly found in Fig. 8. The AUCs of the DensNet-BC1615, DensNet-BC169 and the ensemble classifier were 0.9968, 0.9965, and 0.9974, respectively. The reason for the increase in the overall accuracy of the ensemble classifier is that the ensemble classifier actually can classify more samples correctly based on the result of two separate models. For example, one sample gets wrong identification by one model but obtains the correct label in another model, in this case, the ensemble classifier can eventually return the correct classification result to this sample. As a result, more samples can get correct classification by using an ensemble classifier.

### The real-time automatic identification system

**Figure 9:**
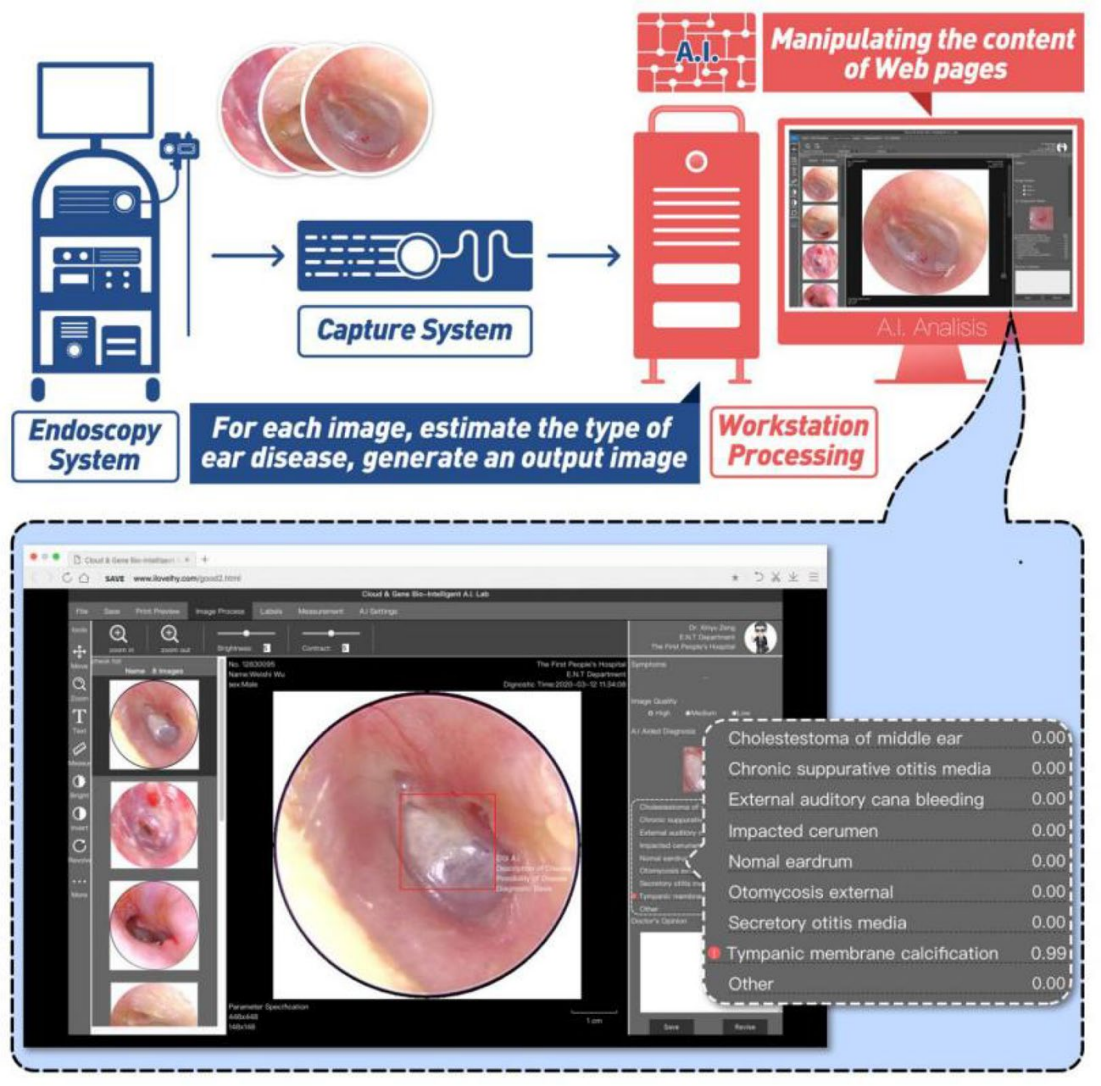
A schematic diagram of the real-time automatic identification system for ear diseases and the system web page of the system. The red border is drawn manually by the doctor.

A real-time automatic identification system was designed by combining an endoscopy system and ensemble classifier, as shown in Fig. 7, and its web page also can be seen in. The image was obtained from the endoscopy system by adjusting the focus. The image got into the real-time recognition system after being processed by the capture system, and then the prediction results were obtained by the ensemble classifier, which was displayed on the web page of the system. Provided that an image is in the process of real-time classification if the maximum probability of the eight classes (NE, CME, CSOM, EACB, IC, OE, SOM, TMC) estimated by one of the three models (DenseNet-BC169, DenseNet-BC1615 and ensemble classifier) is less than or equal to 0.3, or more than three categories of the prediction probabilities are greater than 0.3 in any model of the three, the system would point out that the image is likely to be excluded in those eight categories and give tips on the web page of the system (others, as shown in the inset of Fig. 9), which will arouse doctors' attention and give artificial intervention to avoid misjudgment.

**Figure 10:**
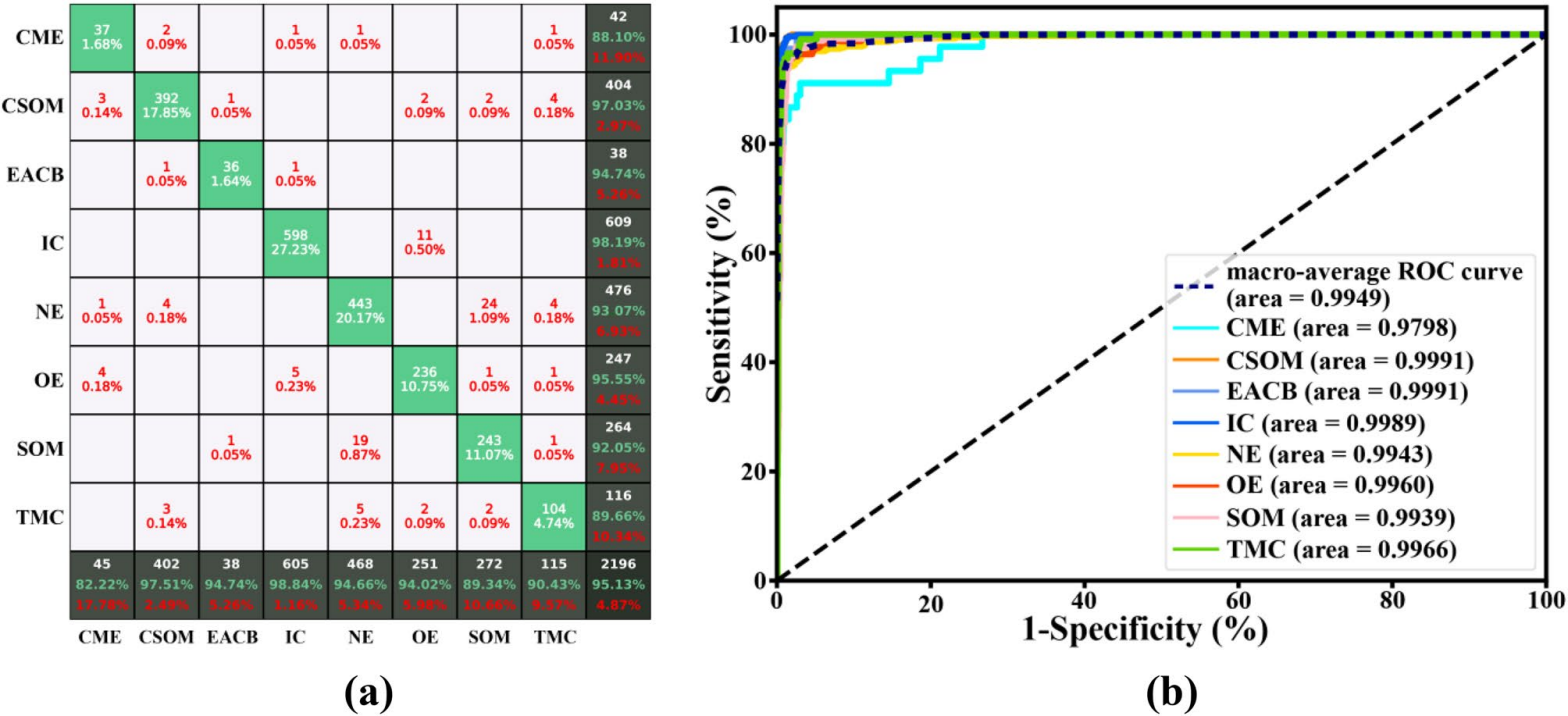
The Confusion matrices (**a**) and sensitivity–specificity (**b**) curve of the real-time automatic identification system based on the ensemble classifier at endoscopic images about the past six months.

In addition, to test the working stability of the real-time automatic identification system, we do a general analysis of the confusion matrices and the sensitivity–specificity curve using the clinical endoscopic images of the department of otolaryngology in the people's hospital of Shenzhen Baoan district in the past six months, as shown in Fig. 10. The average classification accuracy and the AUCs were 95.127% and 0.9949, respectively. The actual test shows that the system achieves the desired objective and is stable and reliable. The response forecast time of the system is about 0.7 s for one endoscopic image.

## Discussion

Diagnosis of ear disease mainly depends on otoscopy and physician's experience^29^, especially external auditory canal. For the accuracy of the ear disease diagnosis, otolaryngologists executed demonstrably better than pediatricians and general practitioners, because diagnosis by otoendoscopy needed expertise in otology doctors^30^. In this condition, the deep network model could provide physicians with suggesting potential diagnosis according to otoendoscopic image^8,9,31^.

In the current model, a dataset of 20,542 labeled otoendoscopic images from more than 40,000 patients was used. Otoendoscopic images of the eardrum and external auditory canal were classified into six categories. The classification accuracy of the current model has reached 95.59%. Previous studies make use of 10,544, 389 and 391 images to analyze ear diseases, with the accuracy of 93.73%, 86.84% and 80.6%^31–33^, respectively. Unluckily, this is only for scientific research and has not been translated into applications. In our work, we also build a real-time interactive detection system that provides doctors with real-time diagnostic results for complementary medical benefits. The classification accuracy of our model reached 95.59%.

There are a few notable limitations to this study. These include the collection of the video frame by a simple operator who is also an acknowledged expert in otology and the use of video frame recordings instead of real-time assessments of ear disease. Nevertheless, we hope that the competence to operate and stabilize the instrument to let stable imaging of the eardrum and external auditory canal in focus will be obtainable by an otoscope inspector with a generally-used skill. Even though this is not the real-time detecting of the eardrum and external auditory canal in clinical treatment because this model has not yet been utilized in an actual patient deployment scenario, as is mentioned above, the testing dataset is untouched, raw and our model executes in almost real-time.

Looking ahead, an approach similar to deep learning also has great potential in improving endoscopic otologic diagnosis. In addition, alternative endoscopic images, confocal laser and endocytoscopy, for example, are most likely to be utilized to train this platform to supply an explanation of clinically acquired images.

In summary, this study shows that a real-time deep learning system of ear disease diagnosis according to otoscopy can realize high accuracy in sorting the disease of the eardrum and external auditory canal when used on unaltered otoscopy, video sequences. Given its high accuracy, we plan to conduct clinical trials to assess the potential of this imaging analytics artificial intelligence model in day-to-day practice. For possible future works, we plan to adapt our method to other kinds of medical images like rhinoscopes or laryngoscopes. Besides, we also plan to develop our method to desktop software, which could be a very useful aided tool for the diagnosis of ear disease.
